# At-C-RNA database, a one-stop source for information on circRNAs in *Arabidopsis thaliana* in a unified format

**DOI:** 10.1093/database/baab074

**Published:** 2021-11-11

**Authors:** Katarzyna Nowis, Paulina Jackowiak, Marek Figlerowicz, Anna Philips

**Affiliations:** Institute of Bioorganic Chemistry, Polish Academy of Sciences, Z. Noskowskiego str. 12/14, Poznan 61-704, Poland; Institute of Bioorganic Chemistry, Polish Academy of Sciences, Z. Noskowskiego str. 12/14, Poznan 61-704, Poland; Institute of Bioorganic Chemistry, Polish Academy of Sciences, Z. Noskowskiego str. 12/14, Poznan 61-704, Poland; Institute of Bioorganic Chemistry, Polish Academy of Sciences, Z. Noskowskiego str. 12/14, Poznan 61-704, Poland

## Abstract

Circular RNAs (circRNAs) are a large class of noncoding RNAs with functions that, in most cases, remain unknown. Recent genome-wide analysis of circRNAs using RNA-Seq has revealed that circRNAs are abundant and some of them conserved in plants. Furthermore, it has been shown that the expression of circRNAs in plants is regulated in a tissue-specific manner. *Arabidopsis thaliana* circular RNA database is a new resource designed to integrate and standardize the data available for circRNAs in a model plant *A. thaliana*, which is currently the best-characterized plant in terms of circRNAs. The resource integrates all applicable publicly available RNA-seq datasets. These datasets were subjected to extensive reanalysis and curation, yielding results in a unified format. Moreover, all data were normalized according to our optimized approach developed for circRNA identification in plants. As a result, the database accommodates circRNAs identified across organs and seedlings of wild-type *A. thaliana* and its single-gene knockout mutants for genes related to splicing. The database provides free access to unified data and search functionalities, thus enabling comparative analyses of *A. thaliana* circRNAs between organs, variants and studies for the first time.

Database URLhttps://plantcircrna.ibch.poznan.pl/

## Introduction

Circular RNAs (circRNAs) are a class of noncoding alternatively spliced transcripts. It has been shown that circRNAs are present across the eukaryotic tree of life (1). Most efforts have been put into the identification and functional studies of circRNAs in animals (2) and humans (3–7). However, reliable identification and quantitation of plant circRNAs appear to be indispensable not only for the plant science field but also for the proper understanding of the universal rules that govern the formation and functioning of these RNAs across kingdoms and the significance of circRNAs in a broad evolutionary context.

**Figure 1.**
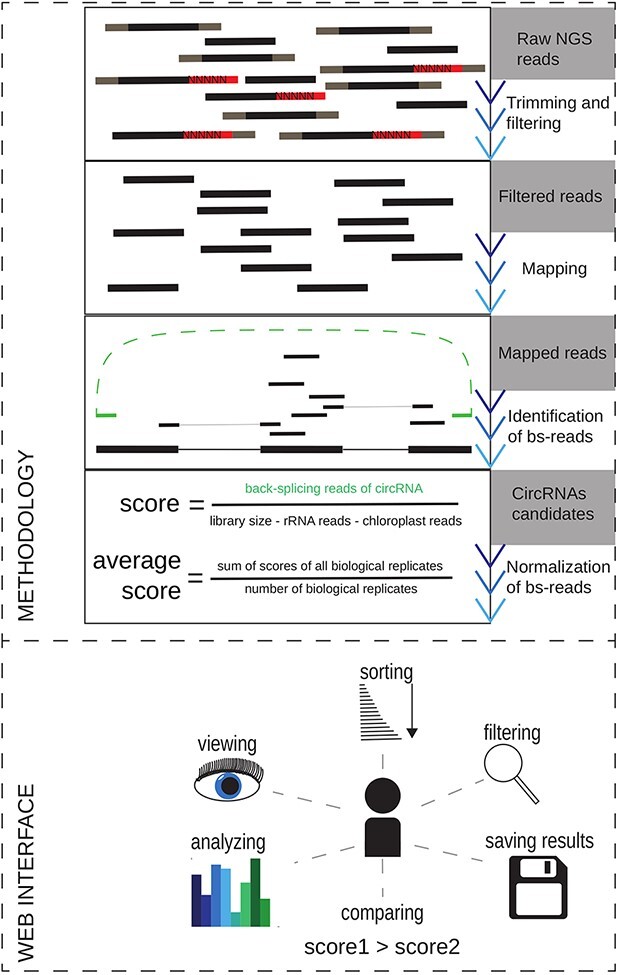
The overview of the methodology used for circRNAs reidentification in the publicly available datasets and the schematic view of the At-C-RNA utilities.

The advent of RNA-Seq has driven the rapid expansion of circRNA studies. Next-generation sequencing technology can provide a comprehensive distribution of circRNAs in the whole organism and its particular organs. This situation is reflected in an increasing number of RNA-seq-based reports on plant circRNAs. Although *A. thaliana* circRNAs have been characterized in multiple studies, a comparison of their results reveals clear discrepancies. The main reason for this situation is a lack of standardization in the methods applied for circRNA analyses. For example, a large fraction of these molecules was identified based on RNA-seq data generated earlier to study gene expression levels. Moreover, the isolation, sequencing and bioinformatics protocols were rarely optimized for circRNA research and differed significantly between studies. This led to the situation that the results published on circRNAs were inconsistent and impossible to comprehensively analyze. The data obtained thus far have been deposited in PlantcircBase (8), which encompasses 19 plant species, including *A. thaliana*, in PlantCircNet (9) and in AtCircDB (10), dedicated exclusively to circRNAs in *A. thaliana*. Unfortunately, circRNAs included in these databases come from different studies and were not curated, nor were their representations unified or normalized. None of these databases include the circRNAs identified in *A. thaliana* knockout mutants. Given the above, comprehensive comparative analyses of circRNAs in this model species have been significantly hampered. To change this situation, we reanalyzed RNA-seq raw data available in the public domain with our protocol for circRNA identification in plants (11) and developed At-C-RNA database to integrate and standardize the available circRNA data (see Figure 1).

## Materials and methods

### Data source

The At-C-RNA database consists of circRNAs identified by reanalyzing publicly available RNA-seq data. CircRNAs do not have polyA tail and thus can only be identified in datasets generated for rRNA-depleted libraries (and not polyA-selected). In search for relevant data, we browsed SRA NCBI with the following criteria: *A. thaliana* species, transcriptomic data, ncRNA, RNA-seq method, Illumina platform, paired-end library layout and rRNA-depleted data (‘reduced representation’ or ‘inverse RNA selection’, according to NCBI guidelines). SRA NCBI search query looked as follows: *‘Arabidopsis thaliana’[Organism] AND‘transcriptomic’[Source] AND (‘rna seq’[Strategy] OR‘ncrna seq’[Strategy]) AND ‘platform illumina’[Properties] AND‘library layout paired’[Properties] AND (‘reduced representation’[Selection] OR‘inverse rrna’[Selection])*. Moreover, we compared the sources used by other plant circRNA databases and we chose those that met our criteria. In total, we utilized third-party data from eight studies (100 SRA files) and two datasets (110 SRA files) from our previous studies (11, 12). All analyzed datasets are presented in Table 1.

**Table 1.** The publicly available datasets utilized by At-C-RNA

BioProject	SRA IDs	Study
PRJNA525820	SRR11279578, SRR11279579, SRR11279580, SRR11279581, SRR11279582, SRR11279583, SRR11279584, SRR11279585, SRR11279586, SRR11279587, SRR11279588, SRR11279589, SRR11279590, SRR11279591, SRR11279592, SRR11279593, SRR11279594, SRR11279595, SRR11279596, SRR11279597, SRR11279598, SRR11279599, SRR11279600, SRR11279601, SRR11279602, SRR11279603, SRR11279604, SRR11279605, SRR11279606, SRR11279607, SRR11279608, SRR11279609, SRR11279610, SRR11279611	(11)
PRJNA630951	SRR11784202, SRR11784203, SRR11784204, SRR11784205, SRR11784206, SRR11784207, SRR11784208, SRR11784209, SRR11784210, SRR11784211, SRR11784212, SRR11784213, SRR11784214, SRR11784215, SRR11784216, SRR11784217, SRR11784218, SRR11784219, SRR11784220, SRR11784221, SRR11784222, SRR11784223, SRR11784224, SRR11784225, SRR11784226, SRR11784227, SRR11784228, SRR11784229, SRR11784230, SRR11784231, SRR11784232, SRR11784233, SRR11784234, SRR11784235, SRR11784236, SRR11784237, SRR11784238, SRR11784239, SRR11784240, SRR11784241, SRR11784242, SRR11784243, SRR11784244, SRR11784245, SRR11784246, SRR11784247, SRR11784248, SRR11784249, SRR11784250, SRR11784251, SRR11784252, SRR11784253, SRR11784254, SRR11784255, SRR11784256, SRR11784257, SRR11784258, SRR11784259, SRR11784260, SRR11784261, SRR11784262, SRR11784263, SRR11784264, SRR11784265, SRR11784266, SRR11784267, SRR11784268, SRR11784269, SRR11784270, SRR11784271, SRR11784272, SRR11784273, SRR11784274, SRR11784275, SRR11784276, SRR11784277	(12)
PRJDB6099	DRR099080, DRR099081, DRR099082, DRR099083, DRR099084, DRR099085	NA
PRJNA218215	SRR1004790, SRR1004791, SRR1004829, SRR1004830, SRR1004831, SRR1004832, SRR1004833, SRR1004834	(14)
PRJNA596364	SRR10727148, SRR10727149, SRR10727150, SRR10727151, SRR10727152, SRR10727153, SRR10727154, SRR10727155, SRR10727156	(15)
PRJNA186843	SRR2079771, SRR2079772, SRR2079773, SRR2079774, SRR2079775, SRR2079776, SRR2079777, SRR2079778, SRR2079779, SRR2079780, SRR2079781, SRR2079782, SRR2079783, SRR2079784, SRR2079785, SRR2079786, SRR2079787, SRR2079788, SRR2079789, SRR2079790, SRR2079791, SRR2079792, SRR2079793, SRR2079794, SRR2079795, SRR2079796, SRR2079797, SRR2079798	(16)
PRJNA311178	SRR3151787, SRR5591021, SRR5591022, SRR5591023, SRR5591024, SRR5591025, SRR5591026, SRR5591027, SRR5591028, SRR5591029, SRR5591030, SRR5591031, SRR5591032, SRR5591033	(17)
PRJNA437291	SRR6814509	(18)
PRJNA511671	SRR8368644, SRR8368646, SRR8368647, SRR8368649, SRR8368650, SRR8368652, SRR8368653, SRR8368635, SRR8368636, SRR8368637, SRR8368638, SRR8368639, SRR8368640, SRR8368641, SRR8368642, SRR8368643, SRR8368645, SRR8368648, SRR8368651, SRR8368654	(19)
PRJEB32782	ERR3489926, ERR3489927, ERR3489928, ERR3489929, ERR3489930, ERR3489931, ERR3489932, ERR3489933, ERR3489934, ERR3489935, ERR3489936, ERR3489937, ERR3489938, ERR3489939	(20)

### Web server implementation

The website was developed in an easy-to-use format with a responsive interface using bootstrap 4, jquery, and CSS technologies. The web framework was designed in Django (python 2.7.15). For table representation jsgrid-1.5.3, select2 was used. Charts showing data from the tables were created with the Google Charts tool and jvenn (13). Excel reports were generated using python packages xlswriter, pandas and NumPy.

## Results

### At-C-RNA content

Currently, in At-C-RNA, 113 327 circRNAs are deposited. Notably, only 19.7%, 18.9% and 16.2%, are reported in PlantcircBase (8), PlantcircNet (9) and AtCircDB (10), respectively (access: 2 September 2021). Each circRNA was assigned a unique identifier according to the common pattern AT_chromosome_number:circRNA_start-circRNA_stop, making comparisons between studies/other databases possible and convenient. The following information is available for each circRNA: (i) the study in which the datasets were generated, (ii) plant line and organ in which circRNAs were identified, (iii) the average score, computed for all circRNAs according to the same procedure, (iv) the individual component scores and (v) information regarding whether a circRNA was confirmed with RNase R experiments. It is worth mentioning that we introduced a novel ‘reproducibility’ criterion in At-C-RNA, as in our previous studies, and we showed that most circRNAs in *A. thaliana* are produced spontaneously and thus possibly carry no biological function (11). It is important to highlight the reproducible circRNAs, which may have functional potential and thus are especially important to the wide range of studies. We defined that a circRNA is reproducible if it was identified in at least four biological replicates in a specific organ and line within a study.

**Figure 2.**
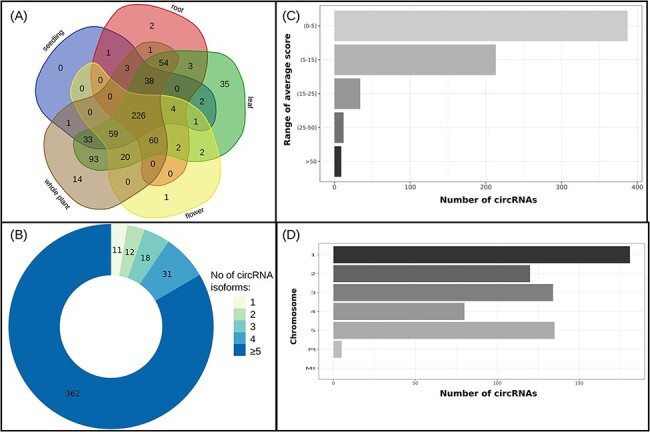
(A) Distribution of reproducible circRNAs across organs/seedling and the whole plant, (B) circRNAs’ isoform number of genes producing reproducible circRNAs, (C) average score ranges for reproducible circRNAs and (D) distribution of reproducible circRNAs across chromosomes.

In total, 655 circRNAs were classified as reproducible (see Figure 2A). Of these, 226 were identified in all of the analyzed plant tissues (flower, leaf, root and seedling) and the whole plant. The highest number of unique reproducible circRNAs (35) was found in the leaf. On the contrary, two other organs, root and flower, revealed only two and one circRNAs typical only to this tissue, respectively. No unique circRNA was found in the seedling. Most of the genes (362) giving rise to the reproducible circRNAs produced above five circRNAs isoforms and only 11 genes produced one circRNA isoform (see Figure 2B). Most reproducible circRNAs (91.6%) score ranges from 1 to 15 what corresponds to the rather low abundance, which most circRNAs display. Fifty-five of circRNAs (8.4%) exceeded an average score over 15 (see Figure 2C). The distribution of reproducible circRNAs on the chromosomes is shown in Figure 2D. Most reproducible circRNAs originated from genes located on chromosome 1 and none from mitochondrial.

### At-C-RNA database utility

#### Novelty

At-C-RNA is the only resource where data across different studies have been reanalyzed, standardized and unified. Moreover, At-C-RNA is the only database that provides information on circRNA reproducibility and occurrence in both wild-type and mutant *A. thaliana* plants.

#### Data browsing and filtering

The At-C-RNA database aggregates circRNAs in a table that allows the user to define the filtering criteria. Data sorting by each column is possible. Each column has a window where filtering criteria can be typed. There is also a possibility to manually delete selected circRNAs from the table. Moreover, users can also filter the table by clicking on the interactive charts below the table.

#### Data download

A previously filtered collection of circRNAs can be downloaded with the ‘Excel Report’ button. Moreover, users can create pivot tables and generate plots from columns of interest.

#### Genomic region and gene information

The database also holds information from external databases (i.e. Ensembl and NCBI). CircRNA ID redirects the user to the Ensembl genome browser where the genomic region of the circRNA of interest can be explored. Moreover, users can read extended information about genes using an external link to NCBI.

#### Common use cases

A frequent task is to search for circRNAs that are produced in a reproducible manner, as only such molecules may carry biological functions. At-C-RNA is the only database where data were curated, and a reproducibility measure was defined for each circRNA. A default filter on the table enables the visualization of these reproducible circRNAs. Moreover, our database enables multilevel filtering, for example, users can filter the data table showing only reproducible circRNAs from a specific gene that are confirmed in RNase R-treated samples.

#### Data curation

All circRNAs deposited in At-C-RNA were reidentified from raw data by our in-home protocol developed for circRNAs identification in plants. We plan to update the database and successively reanalyze and add new circRNAs data, as they appear in the public domain.

## Discussion

Currently, At-C-RNA is the biggest resource of circRNAs in *A. thaliana*, encompassing 113 327 circRNAs. The At-C-RNA database provides not only a comprehensive and convenient source of unified information on the circRNAs in *A. thaliana* but also a user-friendly interface that allows the user to run analyses, the results of which are available in the form of interactive graphical reports and summaries. This platform can be used in plant circRNA research as well as in all studies that focus on the general features of circRNAs and explore the functional potential of these molecules. By unifying data and providing essential tools, At-C-RNA is a robust platform for comparative analyses of circRNAs. The resource will be curated—we plan to successively reanalyze and add new data from the public domain. We believe that At-C-RNA resources will not only contribute to studies on circRNAs biogenesis and function in plants but also will help to understand the universal rules that govern the formation and functioning of circRNAs and their significance in a broad evolutionary context.

## Supplementary Material

baab074_Supp

## Data Availability

Freely available at https://plantcircrna.ibch.poznan.pl/. Website implemented in Django, MySQL and Apache, with responsive design and all major browsers supported.
